# Blurred Boundaries Between Obsessive and Psychotic Symptoms: A Case Report

**DOI:** 10.1192/j.eurpsy.2025.1717

**Published:** 2025-08-26

**Authors:** F. Mayor Sanabria, C. Regueiro Martín-Albo, M. Fernández Fariña, E. Expósito Durán, M. D. P. Paz Otero, J. Sánchez Rodriguez, B. Serván Rendón-Luna

**Affiliations:** 1 Instituto de Psiquiatría y Salud Mental, Hospital Clínico San Carlos, Madrid, Spain

## Abstract

**Introduction:**

Obsessive-Compulsive Disorder (OCD) and psychotic disorders are traditionally considered distinct entities; however, there is increasing evidence of a spectrum where these conditions overlap. In some cases, OCD presents with poor insight, leading to obsessive thoughts and behaviors that resemble psychotic features. These “schizo-obsessive” phenomena challenge standard diagnostic categories and suggest a continuum between OCD and psychosis, necessitating a more integrated approach to diagnosis and treatment.

We report the case of a 69-year-old male evaluated in the emergency department for severe obsessive symptoms, including intrusive images and compulsive behaviors, accompanied by low insight and depressive symptoms, such as suicidal ideation. Initial management with selective serotonin reuptake inhibitors (SSRIs) led to only partial improvement, highlighting the complexity of distinguishing obsessive from psychotic symptomatology and supporting the concept of a continuum between OCD and psychosis.

**Objectives:**

1. To describe the clinical presentation and management of a patient with OCD and psychotic features.
2. To review the evidence regarding the clinical characteristics and management of the schizo-obsessive spectrum.

**Methods:**

A review of the patient’s clinical history, psychiatric assessments, and treatment responses was conducted. A literature review was also performed to provide an overview of OCD with low insight and schizo-obsessive phenomena.

**Results:**

The schizo-obsessive spectrum concept suggests an overlap between obsessive-compulsive symptoms and psychotic features, particularly when insight is impaired. In OCD with poor insight, obsessions can lose their typical egodystonic quality and appear more like delusions. This challenges traditional diagnostic boundaries and indicates a continuum between OCD and psychosis, where insight fluctuates and symptoms may shift from obsessive to delusional states. Clinical management is complex; combining SSRIs with antipsychotics can be effective, particularly in cases with minimal insight. In our case, the introduction of low-dose aripiprazole led to significant improvement, supporting a combined pharmacological strategy addressing both obsessional and psychotic dimensions and aligning with the schizo-obsessive spectrum framework.

**Conclusions:**

1. This case highlights the difficulty in distinguishing psychotic from obsessive symptoms when insight is poor, emphasizing the need for careful differential diagnosis.
2. The overlap of obsessive and psychotic features in this patient indicates the need for further study of “schizo-obsessive” phenomena.
3. The patient’s positive response to combined SSRIs and antipsychotics suggests this approach may be effective for similar cases with overlapping symptoms.

**Disclosure of Interest:**

None Declared

